# Two major earthquakes in Christchurch were not associated with increased ventricular arrhythmias: Analysis of implanted defibrillator diagnostics

**DOI:** 10.1371/journal.pone.0216521

**Published:** 2019-05-03

**Authors:** Christina Chan, Matthew Daly, Iain Melton, Ian Crozier

**Affiliations:** Department of Cardiology, Christchurch Hospital, Christchurch, New Zealand; Erasmus Medical Center, NETHERLANDS

## Abstract

**Aim:**

Christchurch, New Zealand, experienced two major earthquakes on 4^th^ September 2010 and 22^nd^ February 2011. Previous studies have demonstrated that earthquakes are associated with sudden cardiac deaths. Whilst myocardial ischemia would contribute to this, ventricular arrhythmia triggered by stress has also been suggested. We aim to study the impact of the two earthquakes on ventricular arrhythmia events.

**Methods:**

We conducted a retrospective review of all patients resident in the earthquake zone with implantable defibrillators. Ventricular arrhythmia requiring therapy and non-sustained events were recorded from the period of 30 days before thru 30 days after the two earthquakes. Weekly event rates were calculated and compared using log rank analysis. Results are expressed as mean (range), significance was determined at the <0.05 level.

**Results:**

For the 211 patients who were exposed to the 2010 earthquake, there was no difference in the proportion of patients free of therapy, either Shock or ATP (0.943 before and 0.933 after the earthquake, p = 0.85, ns). Similarly, there was no significant increase in events requiring therapy in the 236 patients exposed to the 2011 earthquake (0.957 before and 0.961 after the earthquake, p = 0.80, ns). We identified one patient who required multiple therapy for ventricular tachycardia immediately following both earthquakes.

**Conclusion:**

The two Christchurch earthquakes were not associated with an increase in the event rate of either sustained or non-sustained ventricular arrhythmias in our patients. We identified only a single patient who had arrhythmic storms immediately following the earthquakes.

## Introduction

Major natural disasters such as earthquakes result in an increased incidence of sudden cardiac death [1, 2]. Whilst some of this is due to myocardial ischemia [1, 2] it has been suggested that stress triggered cardiac arrhythmias contribute to sudden cardiac death associated with major stress [1]. Indeed there are numerous reports of ventricular arrhythmias triggered by stress [3, 4], at least in part by enhanced sympathetic tone [5]. However as these reports are generally case or uncontrolled series [3, 4] the contribution of arrhythmias to the increased sudden death seen in natural disasters remains unclear.

Christchurch, a city of 400,000 persons in New Zealand had not previously experienced a major disaster. On September 4^th^ 2010 at 4 am it was struck by a major earthquake, which resulted in damage to housing, commercial building and the city’s infrastructure though fortunately this event did not result in loss of life. However at 12:51pm on the 22^nd^ of February a second more damaging earthquake occurred. Whilst the earthquake was of only 6.3 magnitude, its epicentre was only 10 km from the city centre, and was very shallow at 5 km. The proximity combined the up thrusting nature of the earthquake resulted in a brief but very violent earthquake with vertical acceleration forces that were up to 2.2 times the force of gravity [6], the greatest vertical acceleration recorded in a New Zealand earthquake. Considerable damage occurred with many buildings collapsing, 6659 people were injured and 182 people died within the first 24 hours [7]. Many homes were uninhabitable or badly damaged and the cities infrastructure was severely damaged with many essentially services lost [8].

These two major earthquakes allowed us to determine the effect of a major population wide stress on the event rate of ventricular arrhythmias as recorded in our patient group with implanted defibrillators that were exposed to these events.

## Methods

The Southern Health and Disability Ethics Committee was consulted for consideration of expedited ethical approval in all earthquake related cardiology audits. They advised ethical approval was not required because our study did not require direct patient contact and would not identify individuals. Given this study was a retrospective audit and the data were analysed and reported anonymously, no consent was required.

We conducted a retrospective review of all patients resided in the earthquake zone with implantable defibrillators. We determined if the patient was exposed to the earthquake by their up to date residential address. We recorded their device diagnostics from the period of 30 days before thru 30 days after the 2 events. All ventricular arrhythmia events recorded by device diagnostics including both sustained events requiring therapy with either shock or anti-tachycardia pacing (ATP) and non-sustained events not receiving therapy were recorded. Weekly event rates were calculated and compared using log rank analysis.

We also identified any patients that had cardiac admissions or arrhythmic storms (2 or appropriate shocks over a 24 hour period) over the study period and analysed the relationship of the arrhythmic storm to the earthquakes. Those with events at the time were confirmed to be in Christchurch at the time by history. Results are expressed as mean (range), significance was determined at the <0.05 level.

## Results

In our region 292 patients are followed with implantable defibrillators. The patients are aged 61.1 (9–88) years and 77% are male, as shown in Table 1.

**Table 1:** Baseline patient characteristics and underlying conditions requiring implanted defibrillators.

Characteristic	Number
Age	61.1 (9–88) years
Male	225 (77%)
ICD for secondary prevention	196 (67%)
**Condition**	
Ischaemic cardiomyopathy	145 (49%)
Idiopathic dilated cardiomyopathy	86 (29%)
Hypertrophic cardiomyopathy	14 (4.8%)
Arrhythmogenic right ventricular cardiomyopathy	4 (1.4%)
Cardiac sarcoidosis	4 (1.4%)
Long QT syndrome	6 (2%)
Brugada syndrome	3 (1%)
Idiopathic ventricular arrhythmia	9 (3%)
Congenital heart disease	4 (1.4%)
Family history of sudden cardiac death	2 (0.7%)

ICD, Impantable cardioverter defibrillator

**Fig 1:**
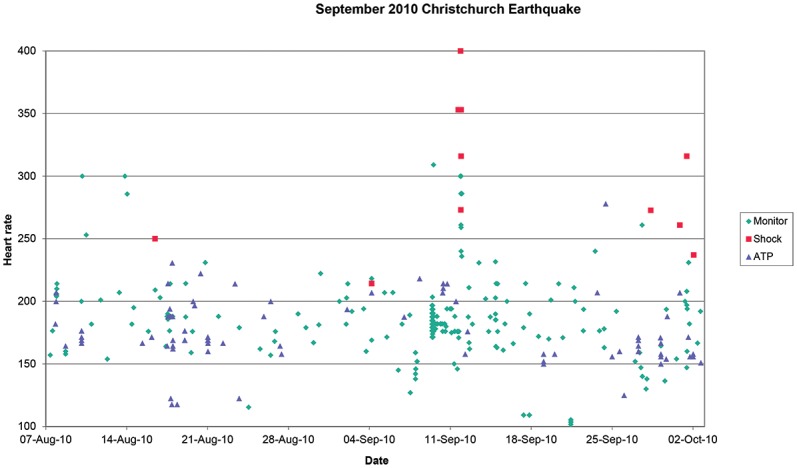
Ventricular arrhythmia events recorded by implantable defibrillators in 211 patients exposed to the earthquake on the 4^th^ September2010. Events are plotted against event heart rate. Events are classified as non-sustained (monitor), sustained treated by shock and sustained treated by antitachycardia pacing (ATP).

Two third had devices implanted for secondary prevention (196 patients). Ischaemic cardiomyopathy was the most common indication for device insertion in 145 patients. This was closely followed by non-ischaemic cardiomyopathy including idiopathic cardiomyopathy, hypertrophic cardiomyopathy, arrhythomogenic right ventricular cardiomyopathy and cardiac sarcoidosis (112 patients). A small number of patients had devices inserted for primary arrhythmic condition such as long QT, Brugada syndrome and idiopathic ventricular arrhythmia (35 patients). We determined that 211 patients were residing in Christchurch at the time of the September 2010 earthquake; events for the period from 30 days before to 30 days after the earthquake are shown in Fig 1. The proportion of patients free of therapy, either Shock or ATP was 0.943 before and 0.933 after the earthquake (p = 0.85, ns).

**Fig 2:**
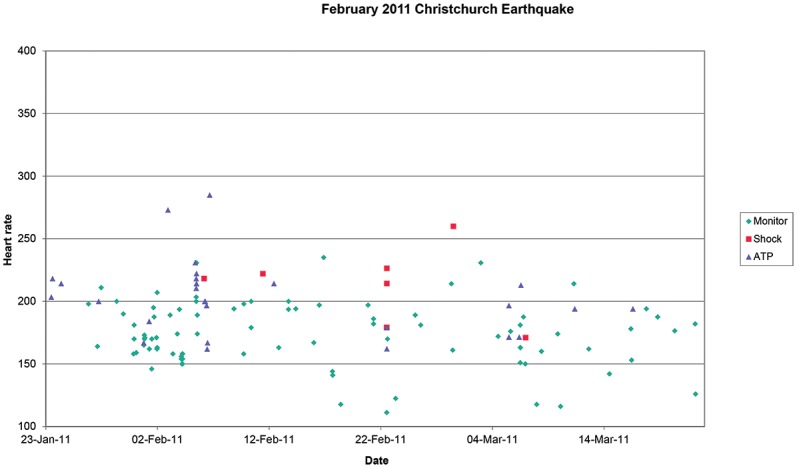
Ventricular arrhythmia events recorded by implantable defibrillators in 236 patients exposed to the earthquake on the 22nd February 2011. Events are plotted against event heart rate. Events are classified as non-sustained (monitor), sustained treated by shock and sustained treated by antitachycardia pacing (ATP).

We determined that 236 patients were residing in the city at the time of the February 2012 earthquake; events for the period from 30 days before to 30 days after the earthquake are shown in Fig 2. The proportion of patients free of therapy, either Shock or ATP was 0.957 before and 0.961 after the earthquake (p = 0.80, ns).

We identified only 2 patients admitted with cardiac events over the study periods, both with arrhythmic storms, and no patients with implanted defibrillators had evidence of an acute coronary syndrome. One patient with a dilated cardiomyopathy and previously stable on medical therapy had multiple episodes of ventricular tachycardia treated with both ATP and shock therapy over several hours immediately following both earthquakes, but was otherwise free of arrhythmia over the study period. We determined that in this case the earthquakes were likely to have triggered ventricular arrhythmias.

The second patient with dilated cardiomyopathy and drug incompliance had an arrhythmic storm 7 days following the September earthquake, associated with hypokalaemia (K 2.7 mmol/L). We determined there was no association between the earthquake and the arrhythmic storm.

The total deaths in Canterbury over the period of 2010 and 2011 were 3708 and 3874 respectively [9, 10]. There was no significant difference between deaths due to ischaemic heart disease in the two years (768 vs 742). Another study done by our department shortly after the earthquakes concluded that natural disasters of this scale triggered significantly more cases of acute myocardial infarction and stress cardiomyopathy but not cardiac arrhythmia or sudden cardiac death [11].

## Discussion

We report the effects of two severe earthquakes on the event rate of ventricular arrhythmias in our patients with implanted defibrillators as determined by the device diagnostics. We found overall that the earthquakes were not associated with an increase in the event rate of either sustained or non-sustained ventricular arrhythmias in our patients. We did however identify a single patient who had arrhythmic storms immediately following both earthquakes.

Our finding of no overall effect on the event rate of ventricular arrhythmias with these two earthquakes was in a population that experienced a marked increase in cardiac admissions and acute ischemic events, and stress cardiomyopathy following the February 2011 earthquake [11].

Our findings are in contrast to the report of increased event rate of haemodynamically unstable ventricular arrhythmias in hospitalised patients with cardiac disease following the earthquakes in Wechuan [12]. Many of these arrhythmias were associated with hypokalaemia, acute myocardial infarction or exacerbations of heart failure.

Our findings also differ from previous reports of stress on life threatening cardiac arrhythmias in patients with implantable defibrillators. Lampert et al in a study of induced ventricular tachycardia in patients with implanted defibrillators reported that mental stress reduced tachycardia cycle length, and rendered the tachycardia less able to be terminated by anti-tachycardia pacing [13]. During the 1994 Northridge earthquake Nishimoto et al reported increased shock therapy in patients with defibrillators [14]. Whilst following the world trade centre attack in 2001 defibrillator patients experienced a 2.3 fold increase in ventricular arrhythmia events during the following 30 days [15].

The reason why we did not observe an increase in event rate of ventricular arrhythmias following the two Christchurch earthquakes, in contrast to other reports of increased ventricular arrhythmias following other major disasters [14, 15] is not immediately apparent. We were able to capture complete data on our population with implanted defibrillators exposed to the earthquake. All devices had electrogram storage so events could be accurately categorised. Also the magnitude of the disaster and the effect of the stress on the population was clear, with a marked surge in stress cardiomyopathy observed following the February 2011 earthquake.

Whilst we only recorded arrhythmias in the small proportion of the population with implantable defibrillators, these patients can be regarded as a sentinel group for arrhythmias in the overall population as they are the patients with the highest risk of ventricular arrhythmias. Therefore we believe our observation casts doubt on the presumption that ventricular arrhythmias in the absence of acute coronary events is a major contributor to the increase in sudden death that is seen in the general population in major natural disasters. This is not to say however that stress and increased sympathetic tone cannot induce life threatening arrhythmias in selected patient groups. Stress is a major precipitant of arrhythmias in catecholamine sensitive arrhythmias such as the long QT syndrome [16], and catecholamine induced ventricular tachycardia [17]. Amongst our patients we had a small number of patients with long QT syndromes, but none experienced arrhythmias. The single patient with arrhythmic storms did not have the long QT syndrome, but clearly experienced arrhythmic storms with both earthquakes. However in the group overall the stress of the earthquakes were not associated with a detectable increase in major ventricular arrhythmias.

Furthermore analysis of the cause for increased sudden cardiac deaths in other earthquakes reveals that these were mostly attributable to acute coronary syndromes. Following the Northridge, California earthquake of 1994 Leor et al reported an increase in sudden cardiac deaths with 24 patients dying on the day of the earthquake compared with a daily average of 4.6. Sixteen of these deaths were ascribed to atherosclerotic cardiovascular disease, and many patients had premonitory symptoms [1]. The average age of patients experiencing sudden death on the day of the earthquake was in the 60s, similar to the preceding week, and not younger patients that would be expected if sudden death was due to recognised conditions known to be associated with stress triggered arrhythmias such as the long QT syndrome. Likewise following the Athens earthquake, the increase in overall cardiac deaths was attributed to atherosclerotic heart disease [2]. These observations are consistent with other major earthquakes following which the myocardial infarction incidence is generally [18, 19], though not always increased [20].

Our study is limited as it is retrospective in nature. However, given that earthquakes are unheralded events, a prospective study design is hard to achieve. Another limitation of this study includes absence of data such medications and co-morbidities. We did not correct for the fact that patients’ response to stress may have been dampened by medications. Finally, we included all patients with defibrillators who had a Christchurch address. It is possible that some were not actually in the city when the earthquakes struck thus underestimate event rates.

## Conclusions

In conclusion we did not observe an increase in ventricular arrhythmia in patients with implanted defibrillators exposed to the two major Christchurch earthquakes. This observation casts some doubts that the increased incidence of sudden death commonly observed during major natural disasters is due to an increase in the rate of primary arrhythmias and suggests that the increased sudden death rate should be ascribed to other mechanisms such myocardial ischaemic events.
